# Training, Pharmacological Ergogenic Aids, Dehydration, and Nutrition Strategies during a Peak Week in Competitive Brazilian Bodybuilders: An Observational Cross-Sectional Study in a Non-World Anti-Doping Agency Competitive Environment

**DOI:** 10.3390/sports12010011

**Published:** 2023-12-29

**Authors:** Fernando Noronha de Almeida, Dahan da Cunha Nascimento, Ronaldo Ferreira Moura, Douglas Leão Peixoto, Wilson Max Almeida Monteiro de Moraes, Brad J. Schoenfeld, Ivo Vieira de Sousa Neto, Jonato Prestes

**Affiliations:** 1 Post graduation Program on Physical Education, Catholic University of Brasilia (UCB), Brasília 71966900, Brazil; fernandyn789@gmail.com (F.N.d.A.); dahanc@hotmail.com (D.d.C.N.); ronaldomoura.personal@gmail.com (R.F.M.); dougleaop@gmail.com (D.L.P.); wmaxnutri@gmail.com (W.M.A.M.d.M.); 2 Department of Exercise Science and Recreation, City University of New York, Herbert H. Lehman College, New York, NY 10468, USA; bradschoenfeldphd@gmail.com; 3 School of Physical Education and Sport of Ribeirão Preto, University of São Paulo (USP), São Paulo 14040900, Brazil; ivoneto04@hotmail.com

**Keywords:** physique, resistance training, competition, androgenic steroids, bodybuilding

## Abstract

Background: this observational and cross-sectional study aimed to describe training, pharmacological ergogenic aids, dehydration, and nutrition strategies during a peak week in competitive bodybuilders and monitor changes that occurred across this period. Methods: Ten amateur bodybuilders were followed during a peak week phase and one day post-contest. Training, diet, dehydration protocol, anabolic steroid use, and nonsteroidal agents were recorded, prescribed, and supervised by the same coach in charge. Body composition, resting metabolic rate, and circumferences were assessed by the research team. Results: Overall, during the peak week phase, the use of anabolic steroids, diuretics, and other pharmacological aids was high among athletes, and the dose and frequency were similar between competitors. Despite the use of supraphysiological doses of drugs, bodybuilders displayed a statistical reduction of lean mass markers, resting metabolic rate, and fat mass markers, possibly influenced by the performance of high volumes of aerobic exercise combined with dietary energy restriction. Conclusions: The main findings of this study display that the coach in charge of Brazilian bodybuilders applied the same anabolic steroid, diuretic, and other pharmacological protocols for all competitors. Moreover, the protocols employed by bodybuilders did not fully attenuate the loss of lean mass during the peak week period.

## 1. Introduction

Bodybuilders are judged on the basis of their aesthetic appearance, which includes components such as muscular mass, symmetry, definition (low body fat mass), and stage presentation [1,2,3]. According to the International Fitness and Bodybuilding Federation (IFBB), “bodybuilding” comprises seven bodyweight categories and includes athletes who do not want to develop their muscles to their extreme full potential but prefer a lighter classic physique [3]. Considering this operational definition, the term “bodybuilder” is used throughout this paper to represent athletes who seek to develop the body’s skeletal muscles in balance and harmony with the primary goal of competing in a physique contest [3].

The bodybuilding season is generally divided into two distinct competitive phases (also known as off-season and pre-season) [2,4]. During the pre-season period, athletes commonly use rigorous nutritional and supplementation interventions (i.e., protein and creatine supplementation), pharmacological aids (i.e., clenbuterol, clomifene, liothyronine, testosterone, nandrolone intramuscular, and various combinations of AASs), dehydration strategies such as reducing water and electrolyte intake to enhance muscle glycogen content and minimize subcutaneous water, and altered training routines (i.e., high weekly aerobic exercise volume) to reduce body fat while maintaining muscle mass [2,5]. However, few studies have evaluated the “peak week” period (i.e., the week prior to the contest) [1,4,6,7] in non-drug-free bodybuilders.

A previous study in natural British bodybuilders demonstrated that carbohydrate and water manipulation were the most prevalent peak week strategies, followed by sodium manipulation and vitamin C loading [1,8,9]. However, drug-free bodybuilders (also known as natural bodybuilders) are known to adhere to World Anti-Doping Agency (WADA) rules, while non-drug-free athletes generally do not [10,11,12]. To this point, there is a paucity of comprehensive data on the topic in competitive, non-drug-free bodybuilders who use polypharmacy strategies to optimize their appearance.

It is commonly believed that bodybuilders are at greater risk of losing muscle mass during the pre-contest phase after engaging in high volumes of aerobic exercise and caloric restriction [13,14]. To counteract the potential loss of muscle during this period, many bodybuilders use anabolic–androgenic steroids (AASs) and nonsteroidal agents (e.g., clenbuterol, liothyronine, clomiphene, human growth hormone, insulin, and diuretics) [2,15,16]. However, the use of these illicit substances in conjunction with water and sodium manipulation accompanied by rapid weight loss might pose a potentially life-threatening risk to the athlete [5,11,17,18,19,20].

In real-world settings, the practices adopted by bodybuilders who use polypharmacy, nutrition, and dehydration strategies lack a scientific-based approach [21]. A previous case report study on six bodybuilders displayed that bodybuilders and coaches were responsible for prescribing their protocols (e.g., polypharmacy) without consultation with other professionals, such as physicians or nutritionists [21]. Also, most of the empirical knowledge gathered is based on the personal trial-and-error experience of coaches in combination with information gleaned from other athletes, coaches, and websites [15,21,22].

Observational and cross-sectional studies can provide real-world information on difficult-to-study topics, thus enhancing practical knowledge for coaches, athletes, nutritionists, and other professionals [23]. To address the gaps in the current literature, this study aimed to describe strategies during the five days pre-contest, contest day, and one day post-contest in non-drug-free bodybuilders supervised by the same coach/trainer in charge. To our knowledge, this study represents a pioneering investigation in assessing changes in anthropometric, body composition, and metabolic outcomes as well as documenting any adverse events in this population across the peak week period. Thus, this observational and cross-sectional study aimed to describe training, pharmacological ergogenic aids, dehydration, and nutrition strategies during a peak week in competitive bodybuilders and monitor changes that occurred across this period.

## 2. Materials and Methods

This was an observational, cross-sectional study that followed the recommendations of the strengthening the reporting of observational studies in epidemiology (STROBE) statement [24]. Thus, the researchers were not responsible for the interventions. The researchers had no role in the individual interventions; data were provided by the subjects and their coach during the peak week. However, body composition, resting metabolic rate, and circumferences were assessed by the research team. This procedure was facilitated by the fact that two of this study’s authors (F.N.A and J.P) were known in competitive Brazilian bodybuilding circles, either as coaches or athletes. Thus, participants were recruited through in-person conversations.

### 2.1. Participants

Ten non-drug-free amateur bodybuilders who participated in either the Amateur South American Arnold Classic or the Brazilian Championship were recruited for the study via convenience sampling. This study was approved by the Catholic University of Brasilia Institutional Review Board (protocol number: 23163019.6.0000.0029). This study was conducted in accordance with the Declaration of Helsinki [25], and all athletes were informed about the study procedures and voluntarily signed an informed consent prior to enrollment.

The inclusion criteria were as follows: (1) competitive male bodybuilders aged 25–60 years of any subdivision (i.e., men’s physique/classic); (2) ability to provide a detailed report of dietary intake, dehydration, AASs use, nonsteroidal agents use, and training program variables during the five days pre-contest, contest day, and one day post-contest. Given the target population, recruitment was resource-constrained, and we made our best efforts to recruit as many participants as possible within practical limitations [26].

### 2.2. Sample Size Calculation

In addition, as this is the first study initiative of this nature at the national (Brazil) level, there were no prior data to support the calculation of the sample size. Therefore, this pilot sample method was used, which consists of generating information about the population on the basis of the first results observed, aiming to obtain a reasonable estimator for the population variance. Also, a sensitivity power analysis, employed when the sample size is already known, was applied with the use of t-tests and means—the difference between two dependent means (matched pairs) [26]. Bonferroni correction was used for sensitivity power analysis with a corrected alpha level of 0.0125 (0.05/four moments), a total sample size of 10, and a power of 0.80 [26,27]. Thus, the minimal statistically detectable effect size to reflect whether a hypothesis test would yield an informative answer based on dietary recommendations for bodybuilding contest preparation of weekly weight loss (% body weight) of 1% was 1.28 Cohen’s d (large effect size) [8,26]. However, considering a reduction of −2.40% (−2.12 kg) body weight between contest day and two days before the contest observed in the present study, the effect size achieved was −1.12 for Cohen’s d (large effect size), with a mean of difference −2.70, an SD of difference of 2.39, a total sample size of 10 participants, an alpha level of 0.05, and a power of 0.88 [27,28].

### 2.3. Study Procedures

**Figure 1:**
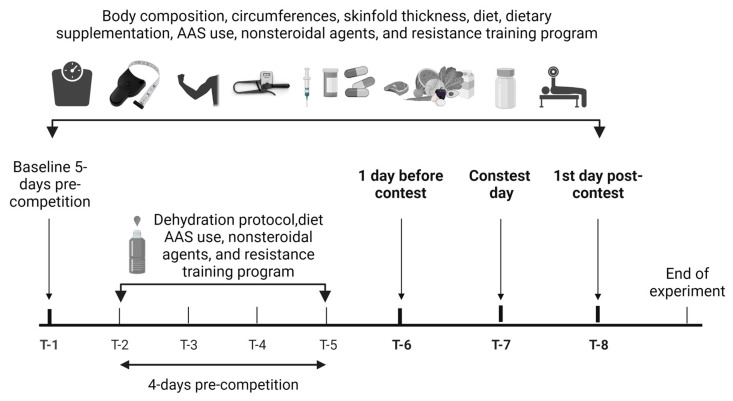
Overview of research design and data collection points. T = a specific moment in time at which an event or measurement occurred.

All measurements were recorded five days before weigh-in, 1 day before the contest day, during the contest day, and on the first day post-contest (Figure 1).

### 2.4. Peak Weak Information

Participants provided the requested information for their peak week preparation and the day following the competition via a questionnaire, which was concluded with measurements of body composition and circumferences by the researcher.

### 2.5. Body Composition and Resting Metabolic Rate

**Figure 2:**
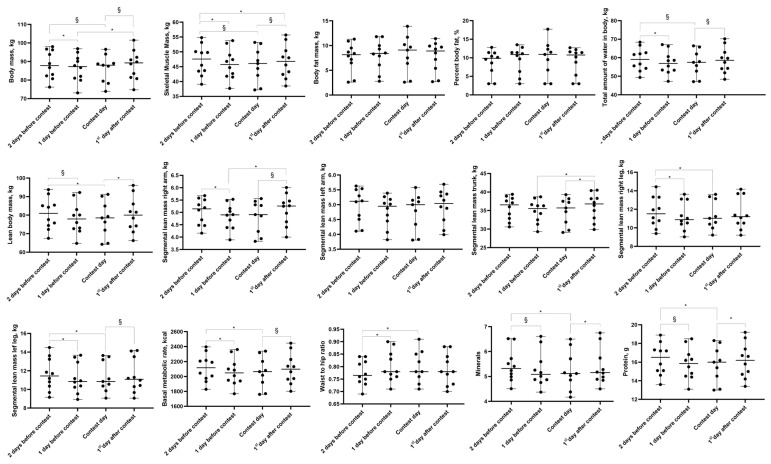
Body composition, protein, and mineral values displayed as means and standard deviations (SDs). Note: * statistically significant difference between moments (*p* < 0.05), § statistically significant difference between moments (*p* < 0.05).

**Figure 3:**
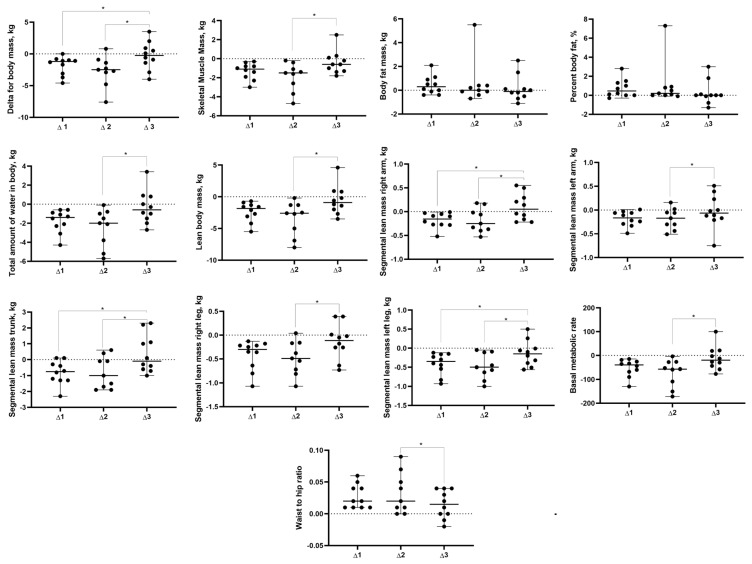
Delta values of body composition variables displayed by mean and standard deviation (SD). One-day variable mean minus two-day variable mean before the contest (Δ1), contest day variable mean minus one-day variable mean before the contest (Δ2), and first day variable mean after the contest minus two-day mean before the contest (Δ3). Note: * statistically significant difference between moments (*p* < 0.05).

Body composition and resting metabolic rate were recorded using multi-frequency bioimpedance analysis (InBody version 270, Cerritos, CA, USA) following the manufacturer’s recommendations to avoid exercising for 6–12 h, eating for 3–4 h, consuming alcohol or caffeine for 24 h, using a sauna, and using lotion or ointment on the hands or feet prior to measurements. It is important to note that multi-frequency bioimpedance analysis displays a high correlation with dual-energy X-ray absorptiometry (DXA) to assess fat mass and lean mass [29,30]. However, given the sample, it was not possible to ensure abstention from diuretic use before the assessment, which might influence the accuracy of the test results. Multi-frequency bioimpedance analysis has also been shown to produce valid estimates of resting metabolic rate when compared with gas analysis [31]. The variables are displayed in Figure 2 and Figure 3 in the results section.

### 2.6. Body Circumference Measurements

Body circumference measurements were obtained using a non-elastic measuring tape (Cescorf™), and skinfold thickness was assessed with calibrated skinfold calipers (Harpenden, Baty International, West Sussex, UK) as per the International Society for the Advancement of Kinanthropometry (ISAK) guidelines [32,33]. Skinfold assessments were conducted on the right side by the same experienced professional (researcher) at seven sites in the following order: subscapularis, triceps, pectoralis, medium axilla, suprailiac, abdomen, and anterior thigh. Skeletal points or landmarks were identified prior to measurements; three measurements were taken, and the median score was recorded [32,33]. Circumference measurements also followed recommendations from ISAK, and the right and left sides were used for the thigh and forearm, respectively. The same procedure was adopted for the waist, hip, and abdominal circumferences with three measurements. The median score was used for statistical analysis [32,33]. Variables are displayed in the Supplementary spreadsheet S1: supplementary DATA and Output S1: DATA_02.

### 2.7. Statistical Analysis

Previous studies suggest that ANOVA is robust when violations of assumptions such as rectangular distribution and exponential distribution are present [34]. The Shapiro–Wilk test for normality was applied, and non-normality was verified for the outcome variables. Also, because of the low sample size and considering that parametric tests are prone to errors if the data contain outliers or are not normally distributed [35,36,37], we employed a non-parametric repeated-measures Friedman’s ANOVA between the four paired conditions and delta analysis with a post hoc test [38]. Because of this, the data are presented as medians and ranges.

To minimize type II error in comparisons, a repeated-measures Friedman’s ANOVA was applied to verify possible differences among deltas (Δ), which were calculated as follows: one-day variable mean minus two-day variable mean before the contest (Δ1), contest day variable mean minus one-day variable mean before the contest (Δ2), and first day variable mean after the contest minus two-day mean before the contest (Δ3). Furthermore, the contest day is when the athlete displays an increase in muscle size due to peaking strategies, such as carbohydrate loading, while pulling subcutaneous water into the muscle, achieving a greater muscle size and a defined appearance for stage presentation [4]. In addition, the day after contest day is a phase in which faster weight gain occurs because of a less rigorous diet and a reduction in training volume and intensity. Thus, these days were used for delta calculations [4]. Also, displaying mean differences between days (Δ) might facilitate sample size calculation for posterior studies. SPSS, JASP, G*Power, and GraphPad were used for statistical analysis. An alpha level of *p* ≤ 0.05 was considered statistically significant for all comparisons [39,40].

## 3. Results

During peak week and the first day post-contest, no potentially life-threatening adverse events were reported by the coach or athletes. Subjects displayed the following baseline characteristics (expressed as median and range): 33.00 (22.00) years of age; 88.90 (21.8) body mass (kg); and 174.00 (22.00) (cm).

### 3.1. Description of the Peak Week Protocol

Participants adopted the same AASs and dehydration protocol as supervised and prescribed by the same coach/trainer in charge. During the 25 days before the contest, the athletes reported using injectable AASs, such as testosterone propionate (150–100 mg 3x/week), trenbolone acetate (100 mg 3x/week), and drostanolone propionate (100 mg 3x/week). However, in the week before the contest, injectable AASs use was changed to AASs pills and included oxandrolone (20 mg 3x daily), stanozolol (20 mg 3x daily), and oxymetholone (50 mg 1x daily). See Table 1 for a detailed description.

For the dehydration protocol, all athletes used the same regressive method of water intake, starting with 10 L per day on the first day and ending with 50 mL of water per meal on the day of the competition. To further facilitate dehydration as well as promote the maintenance of lean mass and fat loss, the athletes used a combination of diuretic drugs and clenbuterol (Table 1).

The diet for the first five days had a higher average amount of protein at all meals and low carbohydrate composition [41]. Two days before the contest, large amounts of carbohydrates were added in addition to proteins at all meals. On the day of the contest, competitors consumed a combination of rice crackers, jelly, processed potato chips, and creatine (Table 1).

The workouts were performed using a split routine, with each muscle group trained once per week, except for the calf muscles, which were trained twice weekly. The athletes performed three exercises in succession for each muscle group in the form of a mini circuit. Four sequences of this circuit were performed in each training session (Table 1). The training volume was relatively low, comprising 12 weekly sets per muscle group. In addition to the resistance training program, the athletes also performed daily steady-state cardiovascular training, with sessions lasting 45 min at ~65% of their maximum heart rate.

### 3.2. Body Mass

Significant time effects were observed for changes in body mass (*p* = 0.001) and delta mean differences (*p* = 0.001). Pairwise comparison identified a statistical decrease in body mass two days before the contest vs. one day before the contest (*p* = 0.037, mean difference of −1.93 kg), contest day vs. two days before the contest (*p* = 0.008, mean difference of −2.70 kg), the first day after the contest vs. one day before the contest (*p* = 0.028, mean difference of 1.56 kg), and the first day after the contest vs. contest day (*p* = 0.006, mean difference of 2.33 kg) (Figure 2). For the delta analysis, a statistical difference was observed between Δ2 and Δ3 (*p* = 0.001, mean difference of −2.33 kg) and Δ1 and Δ3 (*p* = 0.014, mean difference of −1.56 kg) (Figure 3).

### 3.3. Skinfold Thickness and Circumferences

Regarding skinfold thickness measurements, statistically significant time effects were observed for the suprailiac (*p* = 0.031) and abdominal (*p* = 0.044) sites, but the differences became null after pairwise comparisons. A time effect was observed (*p* = 0.009) for the abdominal circumference, and after pairwise comparisons, a statistical difference was observed between contest day and the first day after the contest (*p* = 0.044, mean difference of −1.45 cm). Statistically significant time effects were observed in the circumferences of the right forearm (*p* = 0.004) and the right thigh (*p* = 0.027), but the differences became null after pairwise comparisons. No statistically significant time effects were observed for the other skinfold thickness variables, circumference measurements, or height (Supplementary File).

Statistically significant time effects were observed for the waist/hip ratio (*p* = 0.001) and delta mean differences (*p* = 0.010). After pairwise comparisons, statistical differences were observed between one day before the contest and two days before the contest (*p* = 0.016, mean difference of 0.02) and between contest day and two days before the contest (*p* = 0.011, mean difference of 0.03). For delta, a statistical difference was observed between Δ2 and Δ3 (*p* = 0.040, mean difference of 0.02) (Supplementary File).

### 3.4. Skeletal Lean Mass and Lean Body Mass

Statistically significant time effects were observed for skeletal lean mass (*p* = 0.001) and delta mean differences (*p* = 0.001), and after pairwise comparisons, statistical differences were observed between contest day and the first day after the contest (*p* = 0.003, mean difference of −1.61 kg), between contest day and two days before the contest (*p* = 0.001, mean difference of −1.91), and between two days before the contest and one day before the contest (*p* = 0.037, mean difference of 1.24 kg) (Figure 2). For delta, a statistical difference was observed between Δ2 and Δ3 (*p* = 0.001, mean difference of −1.61 kg) (Figure 3).

Statistically significant time effects were observed for lean body mass (*p* = 0.001) and delta mean differences (*p* = 0.002), and after pairwise comparisons, a statistical difference was observed between contest day and the first day after the contest (*p* = 0.008, mean difference of −3.02 kg), between contest day and two days before the contest (*p* = 0.001, mean difference of −3.38 kg), and between one day before the contest and two days before the contest (*p* = 0.016, mean difference of −2.28 kg) (Figure 2). For delta, a statistical difference was observed between Δ2 and Δ3 (*p* = 0.002, mean difference of −3.02 kg) (Figure 3).

### 3.5. Segmental Lean Mass

Statistically significant time effects were observed for right arm segmental lean mass (*p* = 0.001) and delta mean differences (*p* = 0.001), and after pairwise comparisons, statistical differences were observed between contest day and the first day after the contest (*p* = 0.011, mean difference of −0.31), between one day before the contest and two days before the contest (*p* = 0.037, mean difference of −0.17), and between one day before the contest and the first day post-contest (*p* = 0.006, mean difference of −0.30 kg) (Figure 2). For delta, a statistical difference was observed between Δ2 and Δ3 (*p* = 0.003, mean difference of −0.31 kg) and between Δ1 and Δ3 (*p* = 0.007, mean difference of −0.30 kg) (Figure 3).

Statistically significant time effects were observed for left arm segmental lean mass (*p* = 0.010) and delta mean differences (*p* = 0.016). After pairwise comparisons, the statistical differences became null for the segmental lean mass left arm, but for delta, a statistical difference was observed between Δ2 and Δ3 (*p* = 0.029, mean difference of −0.17 kg) (Figure 3).

Statistically significant time effects were observed for trunk segmental lean mass (*p* = 0.005) and delta mean differences (*p* = 0.002). After pairwise comparison, a statistical difference was observed between contest day and the first day after the contest (*p* = 0.016, mean difference of −1.13 kg) and between one day before the contest and the first day after the contest (*p* = 0.037, mean difference of −1.28 kg) (Figure 2). For delta, statistical differences were observed between Δ2 and Δ3 (*p* = 0.004, mean difference of −1.32 kg) and between Δ1 and Δ3 (*p* = 0.004, mean difference of −1.28 kg) (Figure 3).

Statistically significant time effects were observed for right leg segmental lean mass (*p* = 0.002) and delta mean differences (*p* = 0.020). After pairwise comparisons, a statistical difference was observed between contest day and two days before the contest (*p* = 0.008, mean difference of −0.47 kg) and between one day before the contest and two days before the contest (*p* = 0.021, mean difference of −0.44 kg) (Figure 2). For delta, a statistical difference was observed between Δ2 and Δ3 (*p* = 0.029, mean difference of −0.33 kg). Figure 3.

Statistically significant time effects were observed for left leg segmental lean mass (*p* = 0.001) and delta mean differences (*p* = 0.004). After pairwise comparisons, statistical differences were observed between contest day and the first day after the contest (*p* = 0.037, mean difference of −0.32 kg), between contest day and two days before the contest (*p* = 0.001, mean difference of −0.47 kg), and between one day before the contest and two days before the contest (*p* = 0.003, mean difference of −0.43 kg) (Figure 2). For delta, statistical differences were observed between Δ2 and Δ3 (*p* = 0.029, mean difference of −0.32 kg) and between Δ1 and Δ3 (*p* = 0.029, mean difference of −0.28 kg) (Figure 3).

### 3.6. Total Amount of Body Water

Statistically significant time effects were observed for the total amount of body water (*p* = 0.001) and delta mean differences (*p* = 0.002), and after pairwise comparisons, statistical differences were observed between contest day and the first day after the contest (*p* = 0.006, mean difference of −2.26 kg), between contest day and two days before the contest (*p* = 0.001, mean difference of −2.46 kg), and between one day before the contest and two days before the contest (*p* = 0.021, mean difference of −1.72 kg) (Figure 2). For delta, a statistical difference was observed between Δ2 and Δ3 (*p* = 0.002, mean difference of −2.26 kg) (Figure 3).

### 3.7. Resting Metabolic Rate

Statistically significant time effects were observed for resting metabolic rate (*p* = 0.001) and delta mean differences (*p* = 0.003). After pairwise comparisons, statistical differences were observed between contest day and the first day after the contest (*p* = 0.011, mean difference of −65.77 kcal), between contest day and two days before the contest (*p* = 0.001, mean difference of −73.33 kcal), and between one day before the contest and two days before the contest (*p* = 0.001, mean difference of −50 kcal) (Figure 2). For delta, a statistical difference was observed between Δ2 and Δ3 (*p* = 0.003, mean difference of −65.77 kcal) (Figure 3).

### 3.8. Protein and Mineral Consumption

A statistically significant time effect was observed for proteins consumed (*p* = 0.001), and after pairwise comparisons, statistical differences were observed between contest day and the first day after the contest (*p* = 0.006, mean difference of −0.56), between contest day and two days before the contest (*p* = 0.001, mean difference of −0.68), and between one day before the contest and two days before the contest (*p* = 0.037, mean difference of −0.45) (Figure 3).

**Figure 4:**
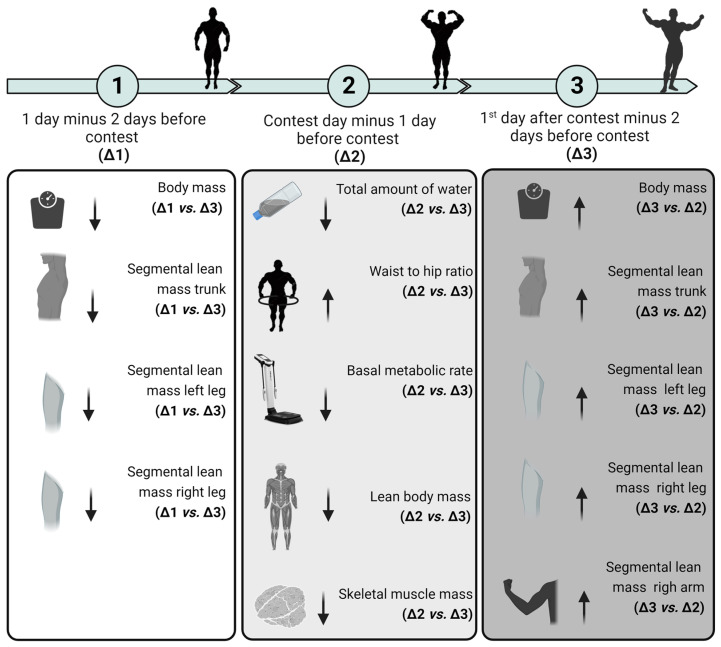
Overview of the findings. One-day variable mean minus two-day variable mean before the contest (Δ1), contest day variable mean minus one-day variable mean before the contest (Δ2), and first day variable mean after the contest minus two-day mean before the contest (Δ3).

A statistically significant time effect was observed for minerals consumed (*p* = 0.001), and after pairwise comparisons, statistical differences were observed between contest day and the first day after the contest (*p* = 0.016, mean difference of −0.18), between contest day and two days before the contest (*p* = 0.001, mean difference of −0.22), and between one day before the contest and two days before the contest (*p* = 0.028, mean difference of −0.13) (Figure 3). For overview of the findings, please refer to Figure 4.

## 4. Discussion

This observational and cross-sectional study aimed to describe training, pharmacological ergogenic aids, dehydration, and nutrition strategies during a peak week in competitive bodybuilders and monitor changes in anthropometric, body composition, and metabolic outcomes as well as document any adverse events across this period. Our findings suggest that bodybuilders can display a reduction in lean mass, resting metabolic rate, hydration, and anthropometric measures up to one day before the contest. The advantages and disadvantages of peak week strategies on competitive performance should be assessed individually, but athletes should be aware of the increased risk of health-related adverse events and side effects associated with the use of polypharmacy use during this critical time.

This study on the practices adopted by bodybuilders corroborated previous findings indicating that most of the empirical knowledge gathered on AASs, dehydration, nutrition, and training prescription is based on the personal trial-and-error experience of the coaches and information gleaned from other athletes, coaches, and websites but not from other professionals, such as nutritionists, physicians, and resistance training specialists [15,21,22]. Also, the coach in charge in this observational and cross-sectional study prescribed the same peak week protocol of dehydration, AASs, and diuretics for all competitors. This “one-size-fits-all” strategy is contrary to the well-established principle of individual differences, which states that the responses to protocols vary between people [42], and it raises the possibility that the associated responses may have been suboptimal for at least some of the competitors.

The athletes in this study employed a supraphysiological polypharmacy strategy during their peak week phase. The approach involved the use of injectable AASs 25 days before the contest and then switching to AASs pills and nonsteroidal drugs 5 days before the contest. This protocol was combined with a dehydration protocol, moderate-volume resistance training, and high-volume cardiovascular training in an effort to maintain their lean mass and minimize body fat percentage. Despite the use of supraphysiological doses of drugs, bodybuilders nevertheless displayed a reduction in markers of lean mass. It should be noted that lean mass measures comprise all non-fat tissue, including body water, which was manipulated during the testing period. Thus, we cannot necessarily extrapolate the findings to changes in muscle mass.

As previously reported, competitive drug-free bodybuilders commonly employ water loading and water restriction protocols [8,9,43,44] similar to those used by the athletes in this study. However, there is a paucity of data on the use of pharmacological aids in non-drug-free athletes during peak week to facilitate dehydration while promoting the maintenance of muscle mass and a reduction in body fat [4,7,21]. Hackett et al. [2] found that the AASs agents most commonly used by non-drug-free bodybuilders during the pre-contest period were stanazolol, boldenone, and oxandrolone, and the most common nonsteroidal drugs were clenbuterol, liothyronine, and clomiphene. In addition, different AASs are used during the off-season phase [2]. However, the type (e.g., injectable and pills), dose, and frequency of use were not reported [2]. Moreover, Gentil et al. [21] investigated the dose and frequency of AASs used during the cutting phase but not specifically the peak week phase in amateur non-free-drug bodybuilders. Athletes reported the use of testosterone propionate, stanozolol, oxandrolone, drostonalone proprianate, and non-steroidal drugs such as ephedrine and hydrochlorothiazide [21].

As previously reported by other studies [2,21] and confirmed with the present results, the AASs protocol used by competitors changes from the bulking phase to the contest phase. This strategy is based on the rationale that the potency of one anabolic agent might be enhanced when consumed simultaneously with other AASs and thus counterbalance the decrease in hormones that occurs in an energy deficit (also known as a “staking” regimen) [13,22,45]. Also, the start of a new cycle with different drugs (e.g., during the peak weak) is intended to increase endogenous testosterone production to prevent the undesirable drop in testosterone concentrations that follows the removal of some AASs after a bulking phase [22].

This study found that one week prior to contest day, AASs pills included oxandrolone (20 mg 3x daily), stanozolol (20 mg 3x daily), and oxymetholone (50 mg 1x daily). The steroid stack was different from that of the previous month, which included injectable AASs, such as testosterone propionate (150–100 mg 3x/week), trenbolone acetate (100 mg 3x/week), and drostanolone propionate (100 mg 3x/week), confirming that athletes engaged in a “staking regimen” [22].

Although AASs are administered by intramuscular injection or oral ingestion, they cross the cell membrane and enter the cell, binding directly to androgen receptors to affect gene expression or undergo bioactivation into dihydrotestosterone [46]. Bodybuilders are judged based on their aesthetic appearance, which includes muscle mass. This physiological process improves protein synthesis and muscle mass, an important component evaluated during stage presentation. Furthermore, athletes and coaches should be aware of probable side effects caused by AASs use, such as gynecomastia, left ventricle hypertrophy, decreased ejection fraction, testicular atrophy, oligospermia, decreased eGFR, hepatotoxicity, clitoral hypertrophy, menstrual cycle disturbances, erythrocytosis, dyslipidemia, and hypertension [46].

The peak week protocol of athletes in this study also included the use of enalapril (an angiotensin-converting enzyme inhibitor drug class that works on the renin–angiotensin–aldosterone system, which is responsible for the regulation of blood pressure and fluid and electrolyte homeostasis [47]), spironolactone (a potassium-sparing diuretic like eplerenone that competitively inhibits mineralocorticoid receptors in the distal convoluted tubule to promote sodium and water excretion and potassium retention [47]), liothyronine (a thyroidal hormone T3 that is normally produced by the thyroid gland [47]), amirolide (a pyrazine compound inhibiting sodium reabsorption [47]), and clenbuterol (a substituted phenylamino ethanol that has beta-2 adrenomimetic properties [47]).

Non-drug-free bodybuilders often use pharmaceutical diuretics to facilitate fluid excretion prior to competition with the goal of minimizing subcutaneous water [9]. The results of this study indicated a significant time effect reduction in the total amount of body water. Although no adverse effects were reported by any competitors in this study, the use of diuretic drugs described herein highlights the dangerous pre-contest practices employed by bodybuilders that potentially pose life-threatening consequences, including an increased risk of hyperkalemia and hyponatremia [17,18].

The athletes in this study also reported the use of clenbuterol for weight loss and the maintenance of lean mass, which can have adverse effects such as pulmonary edema, myocardial infarction, chest pain, palpitations, and anxiety; the coadministration of clenbuterol with drugs such as AASs and thyroid hormones increases the risk of cardiotoxicity [20,48]. However, despite using supraphysiological doses of drugs formulated to maintain skeletal muscle, the bodybuilders in the present study displayed a reduction in markers of lean mass and resting metabolic rate, possibly associated with engagement in high volumes of aerobic exercise and caloric restriction (significant time effect of reduction of protein and mineral consumed before the contest day), as previously reported [2,13,14,21,45].

### Limitations and Strengths of the Study

The present study has several important limitations, including a relatively small sample size of male bodybuilders; the potential for selection bias, which increases the possibility of the inclusion of outliers [23]; the use of the same protocol (i.e., similar training, ergogenic aids, and supplementation) by athletes prescribed by the coach, which is contrary to the well-established principle of individual differences and introduces bias for external validity; and the use of bioelectrical impedance, which is highly influenced by hydration status and thus may not be sensitive to detect subtle changes in muscle mass [49] and was most likely affected by diuretics before body composition evaluation. Moreover, the generalizability of our results is limited due to the men’s physique and classic divisions, the amateur status of the athletes, and the specific time frame of assessment. In addition, whether repeated cycles of peak week strategies over time are detrimental to health status remains undetermined and warrants future investigation. By contrast, this study is one of the few that investigates the strategies used during peak week by non-drug-free bodybuilders.

## 5. Conclusions

In summary, this study indicates that the prescribed peak week protocols for non-drug-free competitors are primarily based on the personal trial-and-error experience of coaches and information gleaned from other athletes, coaches, and websites. Moreover, the results suggest that the polypharmacy protocols employed by the bodybuilders supervised by the same coach did not fully attenuate the loss of lean mass during the peak week period.

## Figures and Tables

**Table 1:** Overview of nutrition, pharmacological ergogenic aids, dehydration, and training program during a peak week.

**Dehydration Protocol and AA Use**														**Note**
	**Sun**	**Mon**	**Tues**		**Wed**	**Thurs**			**Fri** **Weigh-In**			**Sat** **Contest Day**		25 days prior to contest included only injectable AASs, such as testosterone propionate (150–100 mg 3x/week), trenbolone acetate (100 mg 3x/week), and drostanolone propionate (100 mg 3x/week). The last week before the contest included the use of AASs pills, including oxandrolone (20 mg 3x daily), stanozolol (20 mg 3x daily), and oxymetholone (50 mg 1x daily) instead of injectable AASs.
Water, L/per day	10.0	9.0	8.0		6.0	6.0			4.0			0.2		
Enalapril, mg	20.0	20.0	20.0		20.0	20.0			-			-		
Spironolactone, mg	-	12.5	25		50.0	50.0			75.0			150.0		
Liothyronine, mcg	-	25.0	25.0		37.5	37.5			37.5			50.0		
Amiloride, mg	-	-	-		-	-			50.0			-		
Clenbuterol, mg	0.02 three times daily	0.02 three times daily	0.02 three times daily		0.02 three times daily	-			-			-		
**Diet**														**Note**
	**Sun**	**Mon**	**Tues**		**Wed**	**Thurs**			**Fri** **Weigh-in**			**Sat** **Contest Day**		Interval of 3 h between meals.
	Whey/g	Tilapia/g	Salmon/g			Potato/g	Tilapia/g	Salmon/g	Minced meat/g	Rice/g	Dried grape/g	Rice crackers/unit	3 g of jelly	On the Saturday before competition, athletes consumed potatoes, creatine (5 g), Gatorade (200 mL), and a pre-training.
1st meal	60.0	-	-			150.0	150.0	-	80.0	200.0	30.0	4.0	1.0	
2nd meal	-	200.0	-			150.0	150.0	-	80.0	200.0	30.0	4.0	1.0	
3rd meal	-	-	225.0			150.0	150.0	225.0	80.0	200.0	30.0	4.0	1.0	
4th meal	75.0	-	-			150.0	150.0	-	80.0	200.0	30.0	4.0	1.0	
5th meal	-	200.0	-			150.0	150.0	-	80.0	200.0	30.0	4.0	1.0	
6th meal	-	-	225.0			-	-	200.0	80.0	200.0	30.0	4.0	1.0	
**Resistance training program**														
**Frequency**				**Sets**				**Frequency**				**Sets**		
**Upper limbs**								**Lower limbs**						
Once a week and 12 sets of triceps								Once a week and 12 sets of quadriceps						
Once a week and 12 sets of biceps								Once a week and 12 sets of calf						
Once a week and 12 sets of chests								Once a week and 12 sets of posterior thigh						
Once a week and 12 sets of shoulder								Once a week and 12 sets of gluteus						
Once a week and 12 sets of back								-						
Once a week and 12 sets of rectus, oblique abdominal, and paraspinal								-						
**Aerobic training Program**														
Every day, around 65% of the maximum heart rate (HR). Type of exercises used were jogging, elliptical trainer, walking, and cycling.														

## Data Availability

The data from this study can be found in Supplementary File.

## Supplementary Materials

The following supporting information can be downloaded at: https://www.mdpi.com/article/10.3390/sports12010011/s1, Supplementary spreadsheet S1: supplementary DATA; Output S1: DATA_02.
